# A rapid-response ultrasensitive biosensor for influenza virus detection using antibody modified boron-doped diamond

**DOI:** 10.1038/s41598-017-15806-7

**Published:** 2017-11-16

**Authors:** Dawid Nidzworski, Katarzyna Siuzdak, Paweł Niedziałkowski, Robert Bogdanowicz, Michał Sobaszek, Jacek Ryl, Paulina Weiher, Mirosław Sawczak, Elżbieta Wnuk, William A. Goddard, Andrés Jaramillo-Botero, Tadeusz Ossowski

**Affiliations:** 1 Institute of Biotechnology and Molecular Medicine, 3 Trzy Lipy St., 80-172 Gdańsk, Poland; 2 SensDx Ltd, 14b Postepu St., 02-676 Warszawa, Poland; 3 Polish Academy of Sciences, Szewalski Institute of Fluid-Flow Machinery, 14 Fiszera St., Gdańsk, Poland; 4 Department of Analytical Chemistry, Faculty of Chemistry, University of Gdansk, 63 Wita Stwosza St., 80-308 Gdansk, Poland; 5 Department of Metrology and Optoelectronics, Faculty of Electronics, Telecommunications and Informatics, Gdansk University of Technology, 11/12 G. Narutowicza St., 80-233 Gdansk, Poland; 6 Department of Electrochemistry, Corrosion and Materials Engineering, Faculty of Chemistry, Gdansk University of Technology, 11/12 G. Narutowicza St., 80-233 Gdansk, Poland; 7 Materials and Process Simulation Center, California Institute of Technology, 1200 East California Blvd., California, 91125 USA

## Abstract

According to the World Health Organization (WHO), almost 2 billion people each year are infected worldwide with flu-like pathogens including influenza. This is a contagious disease caused by viruses belonging to the family Orthomyxoviridae. Employee absenteeism caused by flu infection costs hundreds of millions of dollars every year. To successfully treat influenza virus infections, detection of the virus during the initial development phase of the infection is critical, when tens to hundreds of virus-associated molecules are present in the patient’s pharynx. In this study, we describe a novel universal diamond biosensor, which enables the specific detection of the virus at ultralow concentrations, even before any clinical symptoms arise. A diamond electrode is surface-functionalized with polyclonal anti-M1 antibodies, which then serve to identify the universal biomarker for the influenza virus, M1 protein. The absorption of the M1 protein onto anti-M1 sites of the electrode change its electrochemical impedance spectra. We achieved a limit of detection of 1 fg/ml in saliva buffer for the M1 biomarker, which corresponds to 5–10 viruses per sample in 5 minutes. Furthermore, the universality of the assay was confirmed by analyzing different strains of influenza A virus.

## Introduction

Multiple methods to enable fast (<2 h) detection of the influenza virus have been developed since the golden standards in virological diagnostics were established – virus isolation from tissue cultures or embryonating chicken eggs. Most of the current methods are based on PCR technology (standard PCR, Reverse Transcription PCR, and real-time PCR) or immunoassays (ELISA tests). In comparison to other molecular methods, PCR technologies offer higher sensitivity and specificity, and are less time-consuming. Yet, they require isolation of the genetic material before analysis, which may lead to sample contamination and expensive instrumentation needs, both of which are considered a major drawback.

To date, different types of biosensors have been developed based on quartz crystal oscillations^1^, surface plasmon resonance^2^, carbon nanotube field effects^3^, imaging ellipsometry^4^, interferometry^5^ or microfluidic^6^. Matsubara *et al*.^7^ recently presented a boron-doped diamond (BDD) electrode terminated with a sialic acid-mimic penta-peptide using click-chemistry reaction, for the detection of influenza virus. The peptide was used instead of the more common sialyloligosaccharide receptor^8,9^, which is the common receptor of influenza A and B. They demonstrated that H1N1 and H3N2 IFVs were detectable using impedance spectroscopy, in the range of 20–400 plaque forming units (pfu) after 45 mins of incubation^7^.

All of above approaches require high complexity analysis equipment or they are time-consuming. Therefore, a simple, versatile, and rapid method for the detection of influenza A virus is needed.

This paper presents an electrochemical nano-scale boron-doped diamond surface sensor specifically functionalized with anti-M1 antibodies for early detection of influenza virus, and constitutes an order of magnitude improvement in detection time (within only 5 mins of incubation) and in resolution (5–10 pfu) when compared to the best previously reported sensor. Our results enable high-precision, high-throughput early detection of the influenza virus from throat or nasal wash specimens. We choose the matrix (M1) protein as a target for detection because it is the only essential viral component for virus-like particles (VLPs) formation and because it is universal to all serotypes of influenza virus.

The BDD electrodes are of great interest, especially in the field of biosensors, since they have enabled the design of new selective and highly sensitive immunosensors^10^. The remarkable electrochemical properties of boron-doped diamond (BDD), including its low background current, wide potential window, high stability combined with biocompatibility, and chemical inertness, makes it a very promising material for the third-generation biosensors^11,12^ combining sensitivity and specificity with the advantages of novel microelectronics. BDD modified electrodes also provide fast electrochemical response^13^.

In this report, the BDD surface was electrochemically functionalized using 4-aminobenzoic acid self-assembled monolayer (SAM), and anti-M1 antibodies were captured onto the SAM. Next, the electrode was immersed in BSA solution to cap open sites and to eliminate unspecific immobilization during measurements. The last step involved introducing the functionalized BDD electrode into a buffer solution with degraded viruses, before measuring the viral concentration. The absorption of the M1 protein onto anti-M1 sites of the electrode change its electrochemical impedance spectra, which is then related to the viral concentration. The multifunctionalization of the BDD electrode surface by antibodies, by electrografting with diazonium salts (Figure S2 in ESI), creates a novel approach for detection of influenza virus on an excellent detection level.

**Figure 1:**
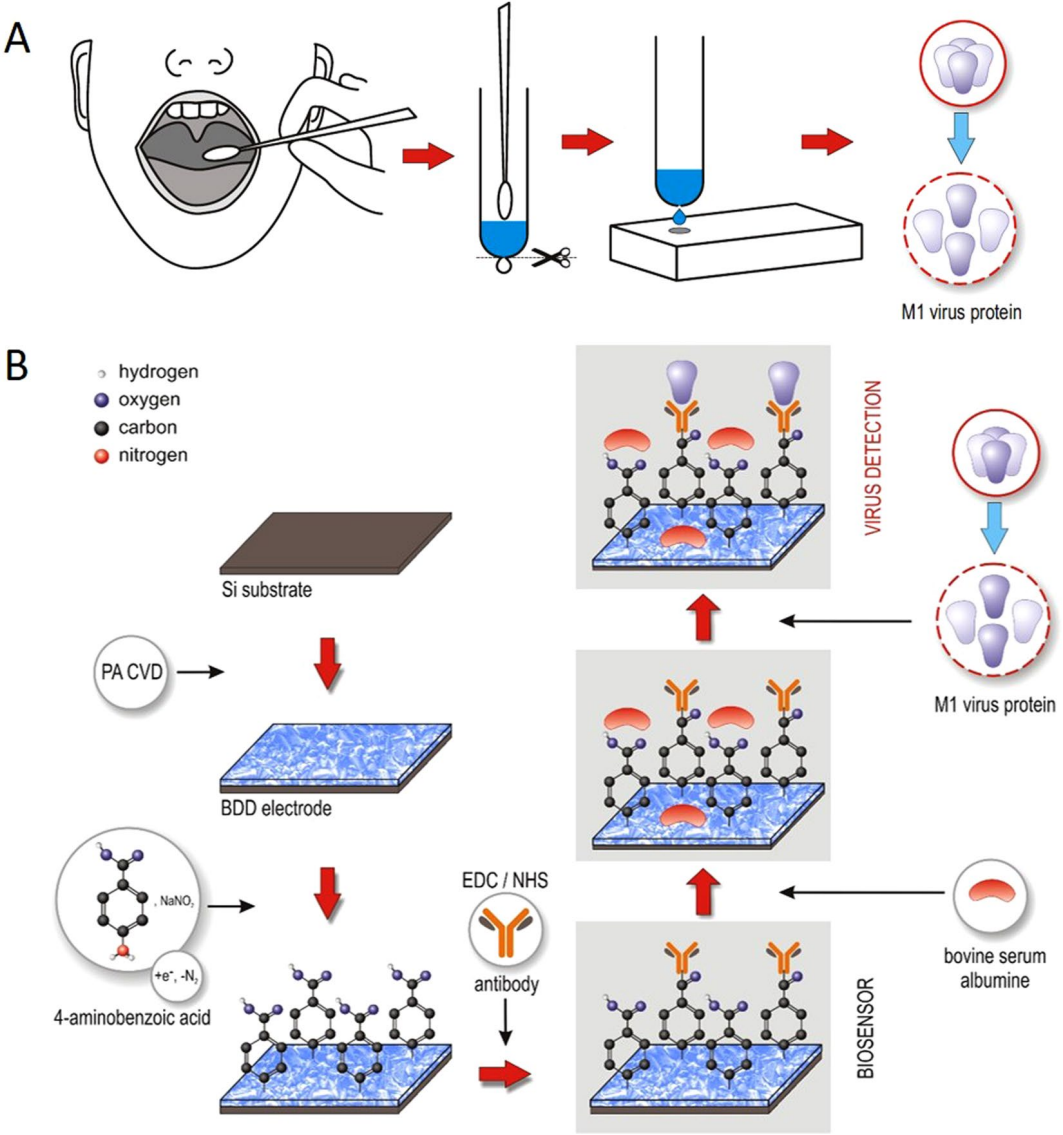
Schematic illustration of biosensing system. (**A**) Throat swab culture acquisition. (**B**) Boron-doped diamond electrode surface modification with polyclonal anti-M1 antibodies, which then serve to identify the universal biomarker for influenza virus, the M1 protein.

The workflow for real-time sample preparation and analysis of influenza infections using the proposed biosensor is illustrated in Fig. 1. A throat or nasal swab culture is acquired in the first step (Fig. 1A). Then, the swab is introduced into a buffer solution containing 0.5% Triton X-100 nonionic surfactant (C_14_H_22_O(C_2_H_4_O)_n_) allowing the virus’ antigen (M1 protein) to be released. About 20–50 µl of the buffer solution is then deposited onto the previously prepared electrode surface (Fig. 1B) and its dielectric properties are measured as a function of frequency using impedance spectroscopy.

## Results and Discussion

Studies by other groups reported grafting of functionalized antibodies to the non-treated bare sensing surface^14^. In this study we implemented the reverse mechanism, i.e., first the functionalization of the sensing surface and then the grafting of the bare untreated antibodies. The use of non-distorted antibodies in our study suggests their interaction in natural environment. Modification of BDD electrodes and subsequent attachment of antibodies was confirmed by X-ray photoemission spectroscopy (XPS). A strong N1s peak derived from amine groups in diazonium salts and antibody aM1 (Figure S3), indicating formation of the antibody modified BDD electrode. A substantial increase in nitrogen originates from the surface modification accompanied by a decrease in the signal from BDD surface. The total share of silicon substrate and PBS buffer residues did not exceed 3% and 1%, respectively. Furthermore, electrochemical modification of the BDD surface by diazonium salts forms a continuous monolayer of carboxyl groups acting as barrier for electric charge transfer (Figure S1) and smoother surface revealed by atomic force microscopy (Figure S3). The antibody–carboxyl interaction induces stereoselectivity of system leading to a high packing density of antibodies and electronic performance of sensor are confirmed by density functional theory (DFT) studies (Supporting Information S2). The electrode stability experiments (Supporting Information S1.3) revealed that the shape of the impedance spectra of the modified electrode is preserved after 24 h of incubation in PBS buffer (Figure S4).

**Figure 2:**
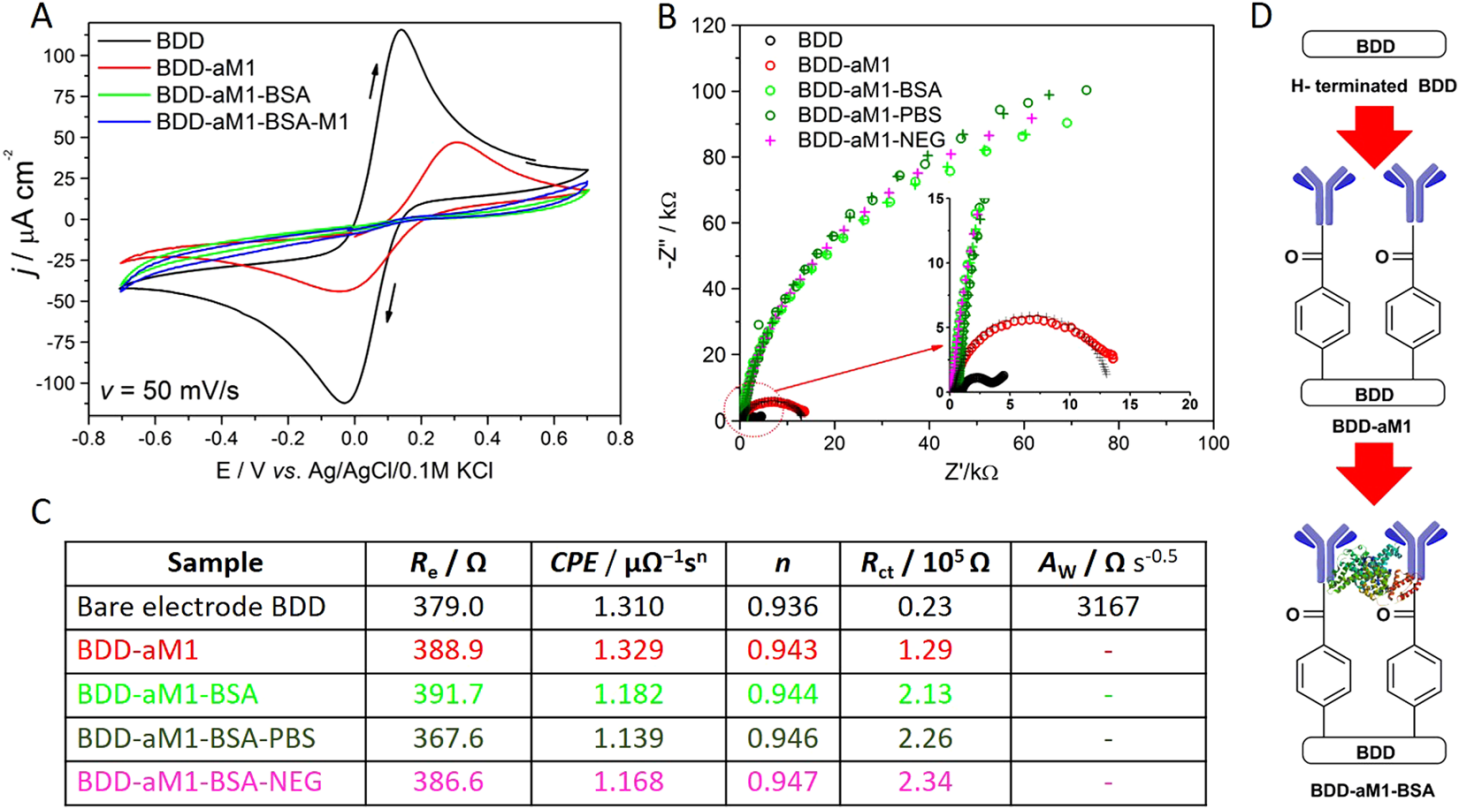
Preparation of surface-modified BDD electrode. (**A**) Cyclic voltammetry curves recorded for bare and modified BDD electrode in 1 mM K_3_Fe(CN)_6_/0.1 PBS with a scan rate of 50 mV/s. (**B**) Impedance spectra of bare and modified BDD electrode after incubation in different solutions recorded in 1 mM K_3_Fe(CN)_6_/0.1 PBS at *E*_f_ =  + 0.13 V vs. Ag/AgCl/0.1 M KCl. (**C**) List of values of elements calculated from the electric equivalent circuit (EEQC) for bare and modified BDD electrode incubated in different solutions. Samples BDD-aM1 – BDD electrode modified with anti-M1 antibody; BDD-aM1-BSA – electrode saturated with BSA; BDD-aM1-PBS – electrode incubated with PBS; BDD-aM1-NEG – electrode incubated with biological samples taken from a healthy man.

Cyclic voltammetry studies were performed to show the changes in the electrode quasi-reversible behavior toward redox active species (Fig. 2A). For bare BDD electrode the difference between oxidation and reduction potential peak equals 0.17 V, whereas when M1 antibodies are bound, the peak current decreases and potential difference grows up to 0.36 V. Incubation of BDD-aM1 electrode in BSA (to avoid overlapping effects beyond the interaction of grafted antibodies) or M1 protein solution, no oxidation or reduction peak was observed. Such a drastic change in CV behavior results from an efficient blockage of electron transfer between electrode and redox active species^15^.

To follow the changes in electrode resistance at each step of electrode modification, the electrochemical impedance spectra (EIS) were recorded at the formal potential of Fe^2+^/Fe^3+^ redox reaction. Nyquist representation of impedance spectra are given for bare BDD surface after its modification with M1 antibody and after saturation with BSA solution. Two other spectra were recorded after incubating electrode only in 0.1 PBS solution (BDD-aM1-PBS) or in a diluted biological sample taken from a swab of a healthy patient (BDD-aM1-NEG) to confirm the lack of influence of neutral buffers/samples to electrode as illustrated in Fig. 2B. According to the shape of the recorded spectra and literature reports^16–18^, collected spectrum for bare BDD electrode was analyzed using an electric equivalent circuit *R*_e_[*CPE*(*R*_*ct*_W)] containing electrolyte resistance *R*_e_, charge transfer resistance *R*_ct_, constant phase element *CPE*, and Warburg element *W* assigned to the diffusional resistance (Fig. 2C). The constant phase element can be viewed as a heuristic method to incorporate the effect of surface heterogeneity both along and through the electrode/electrolyte interface.

Comparing spectra recorded for bare BDD, antibody-modified surface, and after saturation with BSA solution, we may observe a significant increase in resistance *R*_ct_ value. Such an increase in *R*_ct_ suggests successful binding between antigen and bare BDD surface followed by unspecific bonding of BSA to the modified BDD substrate. Anchored M1 antibody and linked BSA in the following step efficiently hampers the charge transfer process resulting in a remarkable increase in *R*_ct_. However, the electrochemical response of BDD-aM1-BSA in the presence of 0.1 PBS or biological sample is unaffected. The change in charge transfer resistance was less than 10% and it was repeatable for all 30 tested BDD-aM1-BSA electrodes comparing the *R*_ct_ values before and after incubation with 0.1 BSA or biological sample. Therefore, such electrodes could be utilized for further investigation as a potential sensor for specific protein presence in analyte.

**Figure 3:**
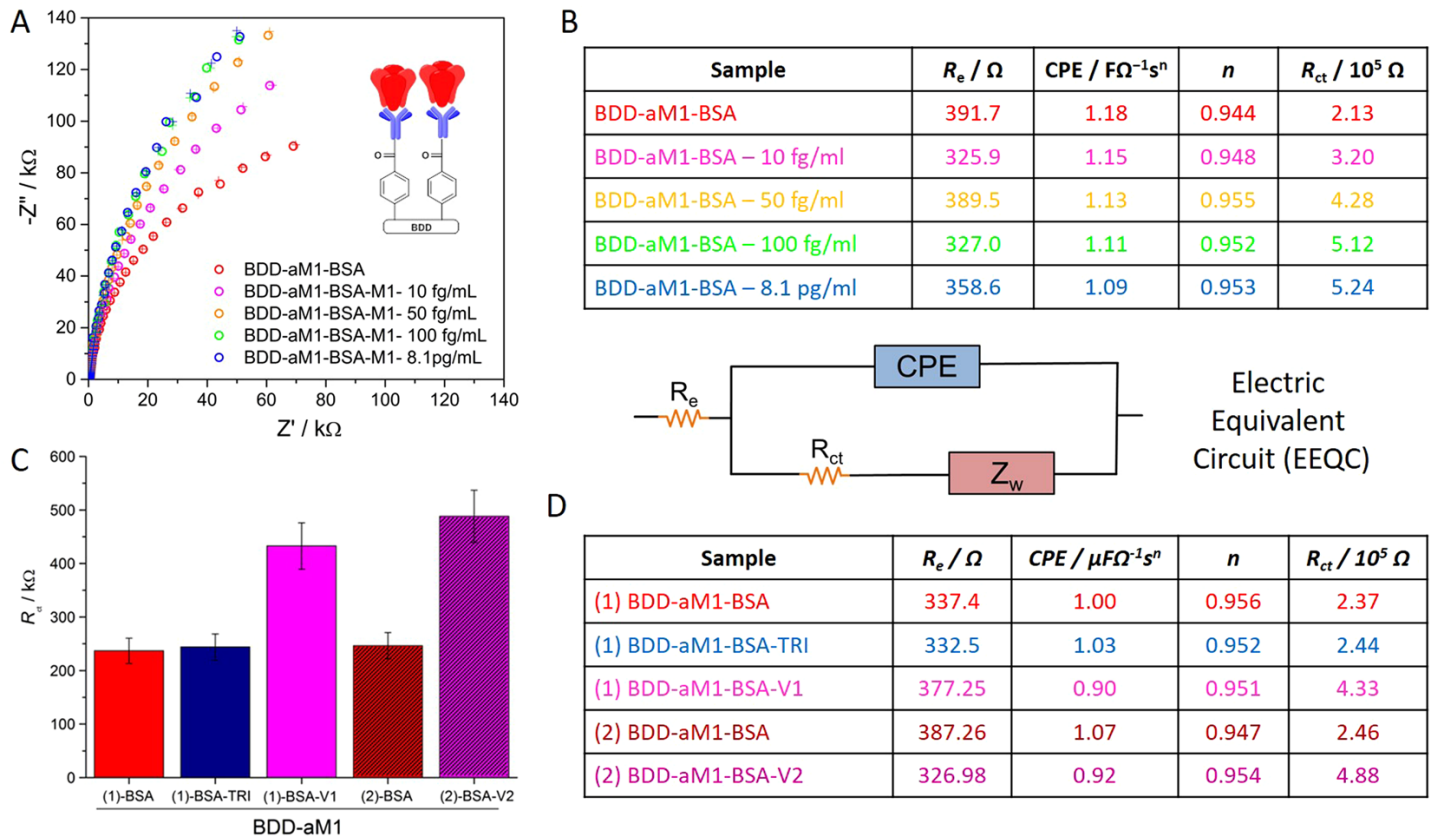
Detection of influenza biomarkers. (**A**) Impedance spectra of BDD-aM1-BSA electrode after incubating in solutions with different M1 concentrations recorded in 1 mM K_3_Fe(CN)_6_/0.1 PBS at *E*_f_ = + 0.13 V vs. Ag/AgCl/0.1 M KCl. (**B**) List of values of elements calculated from the EEQC for BDD-aM1-BSA electrode incubated in different solutions of M1 protein. (**C**) Comparison of charge transfer resistance for two electrodes incubated with H1N1 virus (V1) and H3N2 virus (V2). Error bars show the standard deviations of measurements taken from three independent experiments. Electrochemical species: 1 mM K_3_Fe(CN)_6_ in 0.1 PBS. (**D**) List of values of elements calculated from the EEQC for BDD-aM1-BSA electrode incubated in V1 and V2 in 0.5% Triton X-100/0.1 PBS solution.

The next stage of verification of modified BDD electrode as a potential biosensor includes subsequent incubation in diluted solution of M1 protein and recording the electrode response with electrochemical impedance spectroscopy. Impedance spectra recorded for BDD-aM1-BSA electrode after its incubation with increasing concentrations of M1 protein are presented (Fig. 3A). Figure 3B presents the resistance values with incubation, *R*_ct_ value of electrode changed by 50%, 101%, and 140% when the total concentration of M1 protein solution in contact with the electrode surface was 10, 50, and 100 fg/ml, respectively, compared to the resistance of the electrode when M1 protein is bound to the surface. Such a change is associated with saturation of active antibody sites by the M1 protein, leading to the formation of a dense cover on the BDD electrode. This blocks efficient transfer of electrons between electrode and redox active species present in the electrolyte, which results in an increase in the *R*_ct_ value. Incubation of electrode in more concentrated solution does not change the resistance further due to the lack of active sites^19^ (now blocked by M1 proteins).

The response of these biosensors to different influenza viruses was analyzed by detection of the M1 protein released from the various virus samples (V1 which is H1N1 influenza virus and V2, which is H3N2 influenza virus), buffered in 0.5% Triton X-100/0.1 PBS (see Fig. 3C). The values of each element from EEQC, obtained via fitting, are listed in Fig. 3D. Almost no change was observed in the shape of the EIS spectra upon incubation of the electrode with 0.5% Triton X-100/0.1 PBS. Thus, such a buffer could be used for M1 protein extraction from the virus before applying it on the sensing surface. Furthermore, the R_ct_ value increased by 83% and 98% for V1 and V2, respectively, confirming that the modified BDD electrode quantitatively detects both viruses.

**Figure 4:**
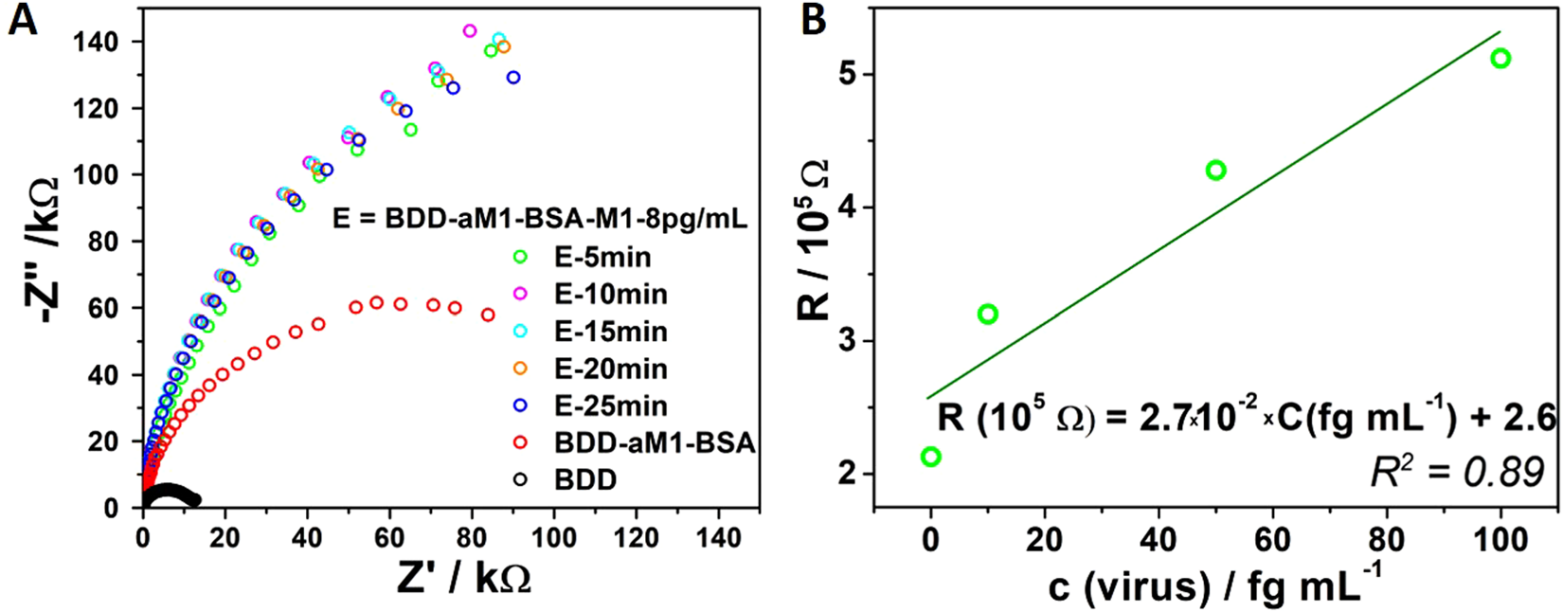
The impedance spectra registered for pristine BDD, BDD modified with aM1-BSA, incubated for different periods (5–25 min) in 8 pg/mL concentrated solution (**A**). The relation between the value of charge transfer resistance and the virus protein concentration (values taken from Table B, Fig. 3) (**B**).

To verify the sensor’s response time, the electrodes were incubated with V1 solution for 5, 10, 15, 20, 25 minutes. As depicted in Fig. 4A, after 5 minutes of incubation, there are no further changes in the spectral shape. This proves that the proposed influenza biosensor has the fastest response, within 5 minutes, when compared to existing technologies. The sensor’s sensitivity and limit of detection are estimated as shown in Fig. 4B. Within the linear range up to 100 fg the sensitivity equals 2.7 kOhm/fg. The limit of detection calculated from the relation LOD = 3 × SD/slope, where SD is the standard deviation in the low concentration range, equals 0.7 fg.

**Figure 5:**
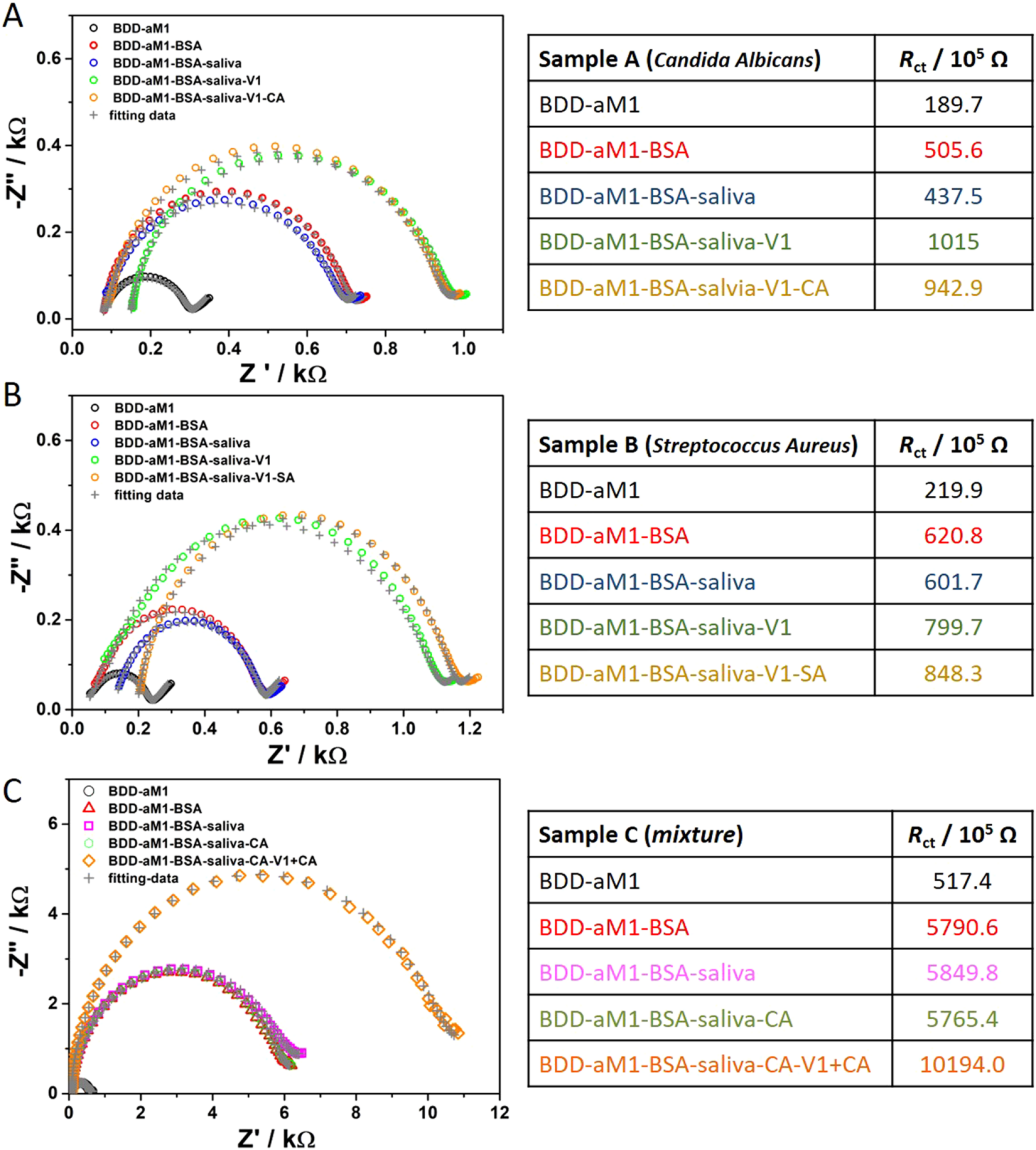
Impedance spectra registered during validation of the biosensors in emulated throat conditions at the formal potential of Fe(CN)_6_^3−/4−^ redox species (after incubation with different interfering pathogens). The tables represent the set of R_ct_ values obtained on the basis of EEQC analysis applied to the impedance spectra for the working electrode after incubation in the different electrolyte conditions. The concentration of S. Aureus, C. Albicans and influenza virus (V1) was kept at 10^6^ cfu or pfu. Abbreviations: V1 - H1N1, SA – *Streptococcus Aureus*, CA- *Candida Albicans*.

To validate the biosensors’ response in a real environment, we emulated throat conditions by incubating the biosensing electrode with various common throat infection pathogens, including with BSA, V1 and species that most commonly lead to human infections such as bacteria *Streptococcus Aureus* (SA) and yeast *Candida Albicans* (CA) in the presence of artificial saliva (see Fig. 5).

The impedance spectra registered at the formal potential of Fe(CN)_6_^3−/4−^ redox species, after electrode incubation in BSA, artificial saliva, H1N1 solution (V1) and separately in SA or CA are shown in Fig. 5A,B. The set of R_ct_ values obtained on the basis of already elaborated EEQC analysis applied to the impedance spectra for each working electrode are shown in the insets of Fig. 5. The response of the biosensor after incubation with the mixture of CA and H1N1 virus solution is shown in Fig. 5C. It is worth noting that, the incubation of the modified electrode with BSA causes R_ct_ increase because of voids in the electrode surface that have no anchored antibodies.

When the electrode was placed into the artificial saliva sample, the space charge resistance does not change significantly, which proves there is no unwanted interaction of the saliva with the biosensing electrode surface. On the contrary, direct coupling of antibodies with the influenza virus proteins (V1 virus solution) affects the shape of resistance and causes a significant increase in the R_ct_. Last but not least, the presence of both bacteria or yeast affect to a large extent the impedance spectra, suggesting no interference. However, after incubation with the mixed V1 virus and CA, the R_ct_ value significantly increases indicating direct interaction between aM1 and virus protein (Fig. 5C).

To summarize, the proposed modified BDD electrode is a valuable influenza-sensing platform exhibiting high selectivity, even in presence of yeast or bacteria cells.

Further tests confirmed the specificity and universality of the antibodies used and thus the whole approach for reliable detection of the influenza virus at a level of 10 fg/mL itself (LOD = 1 fg/ml). Such a low detection limit is unique, when compared to the prior published results on the application of electrochemical sensors designed for specific protein recognition^19,25–27^. These results confirm that our proposed design enables detection of the virus protein in a very small volume and in a very diluted sample, before clinical symptoms appear.

## Conclusions

**Figure 6:**
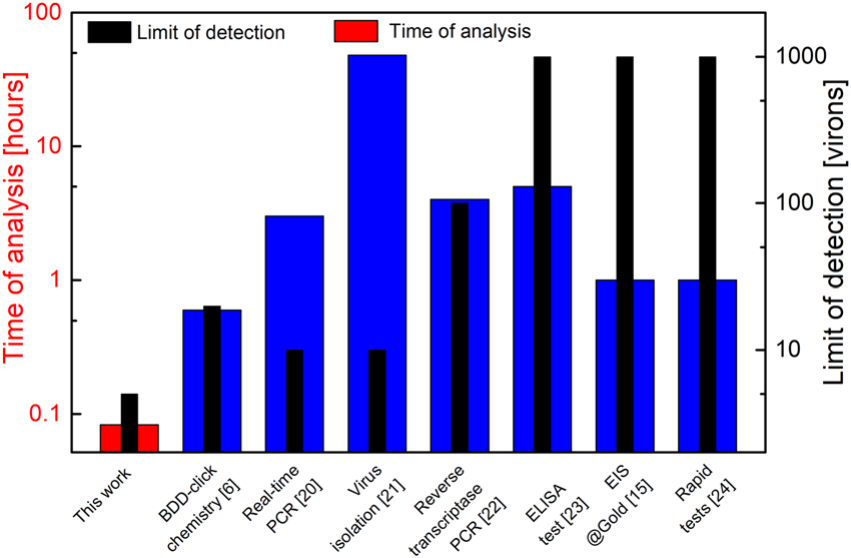
Comparison of parameters of previously reported detection methods of influenza virus versus novel ultrasensitive biosensor presented in this work^7,15,20–24^.

A label-free, ultrasensitive biosensor was designed and characterized for specific detection of the influenza virus. The modified BDD electrodes exhibits high biosensing performance, low background current, large potential window, and extreme stability. Furthermore, our results demonstrate that the proposed method offers significant advantages over existing designs, including: a) short detection and incubation time (<5 min) at room temperature, b) very high sensitivity (LOD 1 fg/ml), d) stability and high repeatability in influenza virus detection, and e) if offers sustained performance, even in the presence of yeast or bacteria cells. The proposed electrode has unspecific response toward buffer solution and un-infected samples from a healthy patient. This method offers the lowest analysis time and the lowest limit of detection compared to the currently used analytical methods (Fig. 6). The change in R_ct_ was less than 10% and it was repeatable for all 30 tested BDD-aM1-BSA electrodes.

We strongly believe that the presented results will lead to major changes in medical diagnostic strategies in the near future, given the wide applicability of this approach involving the use of specific antibodies for selective detection of different biomarkers.

## Methods

### Electrode preparation

The Si/BDD electrodes were synthesized in an MW PA CVD system (Seki Technotron AX5400 S, Japan) on p-type Si wafers with (100) orientation. Substrates were cleaned by sonication in acetone and 2-propanol for 5 min in each solvent. Then, the substrates were seeded by sonication in nanodiamond suspension (crystallite size of 5–10 nm) for 1 hour^11–13^. Finally, the substrates were dried under a stream of nitrogen. The substrate temperature was kept at 700 °C during the deposition process. During the first step, the substrates were etched in hydrogen plasma for 3 min. Excited plasma was ignited with microwave radiation (2.45 GHz). The plasma microwave power, optimized for diamond synthesis, was kept at 1300 W. The gas mixture ratio was 1% of the molar ratio of CH_4_-H_2_ at gas volume of 300 sccm of total flow rate. The base pressure was about 10^−6^ Torr and the process pressure was kept at 50 Torr. All samples were doped using diborane (B_2_H_6_) dopant precursor; [B]/[C] ratio was 10 000 ppm in the plasma resulting in an acceptor concentration of 3 × 10^21^ cm^−3^ ^28,29^. About 6 hours of polycrystalline layer growth resulted in approx. 2 µm thick deposited film.

A four-step pre-treatment of deposited BDD/Si electrodes was applied to obtain H-terminated surface and etch sp^2^ phase impurities. For all Si/BDD samples, the diamond surface was cleaned with acid and hydrogen plasma. First, metallic impurities were dissolved in a hot aqua regia (HNO_3_:HCl/1:3, v-v), followed by the removal of organic impurities by a hot “piranha” solution (H_2_O_2_:H_2_SO_4_/1:3, v-v) at 90 °C. The microwave hydrogen plasma treatment was performed using 1000 W microwave power and 300 sccm of hydrogen gas flow for 10 min. Thus, the BDD surface was made predominantly hydrogen-terminated^30^.

### Modification of the BDD surface by the diazonium salt of 4-aminobenzoic acid

The electrochemical modification of BDD surface electrode was carried out *in situ* in a mixture of diazonium salt of 4-aminobenzoic acid using a BDD working electrode of 8 mm diameter (Figure S1 in ESI). The surface BDD electrode were sonicated before each modification for 5 min in methanol, then electrodes were carefully washed with distilled water and methanol and dried under a stream of air. The functionalization of BDD electrode was performed by the modification procedure described earlier in literature^31,32^.

The 4-aminobenzoic acid (10 mg, 0.073 mmol, Sigma-Aldrich, US) was dissolved in 1 ml of concentrated hydrochloric acid (POCh, Poland). Then 1 ml of water was added while stirring the solution, cooled to 0 °C in an ice bath. After 10 min, 12.5 mg (0.1811 mmol) of sodium nitrite dissolved in 1.5 ml of water was added dropwise to the reaction mixture and stirred in an ice water bath for 30 min. The 0.5 ml diazonium salt of 4-aminobenzoic acid was placed in an electrochemical cell with BDD working electrode and the potential was cycled between 0 to −1.0 V for 10 scans using a scan rate of 100 mVs^−1^. The modified electrodes were washed using distilled water and methanol and dried under a stream of air.

### Specific modification of BDD surface using antibodies

A total of 34.5 mg (0.18 mmol) of 1-Ethyl-3-(3-dimethylaminopropyl) carbodiimide hydrochloride (EDC) (Sigma-Aldrich, US) and 20.9 mg (0.18 mmol) of N-hydroxysuccinimide (NHS) (Sigma-Aldrich, US) were dissolved in 5 ml of dimethylformamide (DMF). One milliliter of this reaction mixture was placed on BDD electrode surface modified with diazonium salt of 4-aminobenzoic acid and left to react for 2 hours, then the solution with antibody concentration of 6 µg/ml diluted in phosphate buffer of pH = 5.5 was added to the electrode and left at 4 °C for 24 hours. The specific modified electrode was washed using only distilled water and dried under a stream of air and used directly for measurements.

The experimental details of applied analytical techniques have been presented in Supporting Information (see Methods in ESI).

### Production of polyclonal anti-M1 antibodies

For antibody production, a rabbit was immunized subcutaneously with 300 μg of purified M1-GST protein mixed with incomplete Freund’s adjuvant in a total volume of 1 ml. The injections were given on day 0 and 21. On day 42, the blood was collected. The obtained sera were tested for purified protein, and optimal dilution of anti-M1 sera for western blot analysis was established as 1:10000. Universality and selectivity of the antibodies were confirmed using western blot analysis. Anti-M1 antibodies were purified by affinity chromatography on CNBr-activated Sepharose 4B coupled with M1-His protein. It was used for fabrication of the biosensor. Efforts were made at all stages of the experiments to minimize the suffering of animals. This study was carried out in strict accordance with the recommendations in the institutional and national guidelines for animal care and use. The protocol was approved by the Committee on the Ethics of Animal Experiments of the Medical University of Gdańsk (Permit Number: 3/2011).

### Viruses propagation

Human influenza A/Virginia/ATCC1/2009 (H1N1) (V1) and A/Aichi/2/68 (H3N2) (V2) were propagated in Madin–Darby canine kidney cells (MDCK) cultured at 37 °C under 5% CO_2_ in Dulbecco’s Modified Eagle’s Medium (D-MEM) (Sigma–Aldrich, US), supplemented with 2 mM L-glutamine, 0.2% bovine serum albumin, 25 mM HEPES buffer, 100 U/ml of penicillin, and 100 µg/ml of streptomycin in the presence of 2 µg/ml TPCK (L-1-tosylamide-2-phenylethyl chloromethyl ketone) − trypsin (Sigma–Aldrich, US). Viral stocks were stored at −70 °C and titrated using plaque assay before use.

### Electrochemical procedures

The cyclic voltammetry (CV) and electrochemical impedance spectroscopy (EIS) were conducted using a PGStat 302N potentionstat-galvanostat system (Methrom, Autolab, Netherlands) in the standard three-electrode assembly, where BDD substrate with different surface modification served as a working electrode. The active surface area has a circular shape with a diameter of 4 mm (geometric surface area of 0.1256 cm^2^).

The Pt mesh was used as a counter electrode, while Ag/AgCl/0.1 M KCl as a reference electrode. All the electrochemical tests were carried out in 1 mM K_3_Fe(CN)_6_/0.1 PBS that was previously deaerated.

To carry out electrochemical tests allowing for the detection of the M1 protein, the adequate chemical surface modification was performed according to the description given earlier to obtain BDD electrode modified with M1 antibody and assigned as BDD-aM1 electrode. To use BDD-aM1 as a biosensing surface, it was incubated for 1 hour at 4 °C with 40 µl of 0.5% concentrated solution of bovine serum albumin (BSA) dissolved in 0.1 PBS solution. BSA molecules bind non-specifically to the aM1-modified BDD substrate^33^, and free host antibody surface is ready for specific anchoring of the antigen. After each incubation with different sample solutions at 4 °C, the electrode surface was washed gently using a fresh 0.1 PBS. Then the electrode surface was placed in contact with the electrolyte solution (100 µl) and the whole experimental setup was conditioned at room temperature. When electrode temperature reached equilibrium with external conditions, the electrochemical measurements were initiated. After each electrochemical measurement in the electrolyte containing ferricyanide redox active species, the electrode surface was washed once again using fresh 0.1 PBS.

Before utilizing the three protocols described later for volume, incubation time, and concentration of protein, a large number of iterations were tested. The present work describes only optimum values under which the best results and comparisons were observed.

The electrochemical impedance spectroscopy measurements were conducted at a frequency range from 20 kHz to 1 Hz, covering 50 points and with 10 mV amplitude of the AC signal. The impedance spectra were collected at the formal potential of the redox reaction determined from redox peak position observed on the cyclic voltammetry curves recorded for bare BDD electrode. Before recording each spectrum, the potential was held for 1 min to achieve a steady-state condition. The impedance data were analyzed based on an electric equivalent circuit (EEQC) using an EIS Spectrum Analyzer program.

### Control measurements

To exclude electrode response to any unspecific detection, the electrode characteristics were tested after substrate incubation in 0.1 PBS and biological material present in the oral cavity of a healthy man. The human biological material was obtained after informed consent from normal donors with the use of procedures approved by the Human Experimentation Committee at Medical University of Gdańsk. Moreover, all methods for humans were performed in accordance with the relevant guidelines and regulations of local the Human Experimentation Committee. First, BDD-aM1-BSA electrode was incubated with 0.1 PBS for 30 min at 4 °C, and after reaching room temperature EIS spectra was recorded. Then, the electrode was washed with fresh 0.1 PBS and incubated for 30 min at 4 °C in 40 µl of 0.1 PBS diluted throat swab from a healthy man. Finally, the electrode response was recorded in the form of impedance spectra.

### Detection of M1 protein

To analyse the electrode sensitivity, the electrode BDD-aM1-BSA was incubated with serial dilution of M1 protein. The protein concentration has been measured by Bradford protein assay. According to the preliminary studies, as a first step BDD-aM1-BSA electrode was incubated in 40 µl of 10 fg/ml M1 protein solution for 30 min at 4 °C. After incubation, the electrode surface was washed with fresh 0.1 PBS and immersed in deareated 1 mM K_3_Fe(CN)_6_/0.1 PBS. When the electrode reached room temperature, EIS spectrum was recorded. Then, the electrode surface was washed with 0.1 PBS and then incubated again in 40 µl of M1 solution, but this time containing 40 fg/ml M1 protein. Thus, the electrode was exposed to 50 fg/ml of protein molecules in total. After incubation for 30 min at 4 °C, the whole experimental procedure, washing with 0.1 PBS and EIS measurement in ferricyanide solution, was repeated. In the following steps electrochemical detection was carried out after subsequent electrode incubation in 40 µl solution of 50 fg/ml concentrated M1 and then finally with 8 pg/ml concentrated M1 protein. Therefore, in the last two steps the total concentration of M1 loaded on the electrode surface was 100 fg/ml and 8.1 pg/ml, respectively.

### Detection of M1 protein from virus suspension

To exclude any impact on the electrode response resulting from Triton X-100 presence, the BDD-aM1-BSA electrode was incubated in 40 µl of 0.5% Triton X-100 in 0.1 PBS for 30 min at room temperature. Subsequently, the electrode was washed with 0.1 PBS and EIS spectra was recorded in the ferricyanide redox solution. The same electrode after rising with 0.1 PBS was incubated for 5 min in 40 µl of V1. Another BDD-aM1-BSA electrode was used for M1 protein released from V2 virus suspended in 0.5% Triton X-100/0.1 PBS solution. Before incubation, the solution of V1 or V2 virus was vigorously shaken. Since the M1 molecular mass is approx. 30 000 Da and ca. 1000 protein molecules create one virus as reported by Ruigrok *et al*.^34^, the 4 × 10^−16^ grams of M1 protein that were detected, correspond to 8 plaque forming units (pfu) of influenza virus.

### Biosensor verification in the presence of *Streptococcus Aureus* and *Candida Albicans*

All the electrochemical tests were carried out in 1 mM K_3_Fe(CN)6/0.1 PBS that was previously deaerated. Artificial salivia was obtained from LC Tech (Germany). The strain *Streptococcus Aureus* was obtained from ATCC collection (ATCC 13420) and *Candida Albicans* was a clinical isolate from Provincial Hospital of Gdansk (17150/2010). After each incubation with different sample solutions at 4 °C, the electrode surface was washed gently using a fresh 0.1 PBS. When the electrode reached room temperature, EIS spectrum was recorded. The electrode was incubated separately in saliva, S. Aureus, Candida A. and H1N1. The concentration of S. Aureus, C. Albicans and influenza virus (V1) was kept at 10^6^ cfu or pfu. Similar concentrations were reported in the patients during inflammation incidents. In the separate experiment, the biosensor was examined by incubation with the mixture od H1N1 virus and Candida A. Briefly, BDD-aM1 was incubated for 1 hour at 4 °C with 35 µl of 0.5% concentrated solution of bovine serum albumin (BSA) dissolved in 0.1 PBS solution. Next, BDD-aM1-BSA electrode was washed with fresh 0.1 PBS and incubated with saliva for 30 min at 4 °C. Then, the electrode was washed with fresh 0.1 PBS and incubated for 30 min at 4 °C in 35 µl of solution containing H1N1 virus suspended in 0.5% Triton X-100/0.1 PBS solution. In the last step, the electrode surface was washed with 0.1 PBS and incubated for 30 min at 4 °C in 35 µl of solution containing *S. Aureus* or *Candida A*. Apart from that, the BDD-aM1 electrode was initially incubated in BSA, artificial saliva and then Candida A. and H1N1 were added to the solution. The mixture was incubated for 30 min at 4 °C followed by EIS spectrum measuring.

The study was carried out in a strict accordance with the recommendations in the institutional and national guidelines for animal care and use. The protocol was approved by the local Committee on the Ethics of Animal Experiments of the Medical University of Gdańsk. Moreover, the human biological material was obtained after informed consent from normal donors with the use of procedures approved by the Human Experimentation Committee at Medical University of Gdańsk.

## Electronic supplementary material

Supplementary Information
