# Supplementary material for: A rapid-response ultrasensitive biosensor for influenza virus detection using antibody modified boron-doped diamond
Source: Sci Rep. 2017 Nov 16;7:15707. doi: 10.1038/s41598-017-15806-7 (PMC5691202; doi:10.1038/s41598-017-15806-7)
Supplement: Supplementary file 1 — Supplementary Information [file 41598_2017_15806_MOESM1_ESM.doc]

**Electronic Supplementary Information**

**A rapid-response ultrasensitive biosensor for influenza virus detection using antibody modified boron-doped diamond**

**Dawid Nidzworski^1,2^, Katarzyna Siuzdak^3^, Paweł Niedziałkowski^4^, Robert Bogdanowicz^5*^, Michał Sobaszek^5^, Jacek Ryl^6^, Paulina Weiher^1^, Mirosław Sawczak^3^, Elżbieta Wnuk^4^, William A. Goddard^7^, Andrés Jaramillo-Botero^7*^, and Tadeusz Ossowski^4^**

*^1^ Institute of Biotechnology and Molecular Medicine, 3 Trzy Lipy St., 80-172 Gdańsk, Poland*

*^2^ ETON Group Ltd, 14b Postepu St., 02-676 Warszawa, Poland*

*^3^ Polish Academy of Sciences, Szewalski Institute of Fluid-Flow Machinery, 14 Fiszera St., Gdańsk, Poland*

*^4^ Department of Analytical Chemistry, Faculty of Chemistry, University of Gdansk, 63 Wita Stwosza St., 80-952 Gdansk, Poland*

*^5^ Department of Metrology and Optoelectronics, Faculty of Electronics, Telecommunications and Informatics, Gdansk University of Technology,* *11/12 G. Narutowicza St., 80-233 Gdansk, Poland*

*^6^ Department of Electrochemistry, Corrosion and Materials Engineering, Faculty of Chemistry, Gdansk University of Technology, 11/12 G. Narutowicza St., 80-233 Gdansk, Poland*

*^7^ Materials and Process Simulation Center, California Institute of Technology, 1200 East California Blvd. California 91125 USA*

^*^ Corresponding author: e-mail: [rbogdan@eti.pg.gda.pl](mailto:rbogdan@eti.pg.gda.pl) and [ajaramil@caltech.edu](mailto:ajaramil@caltech.edu)

**Methods**

**Sensor composition by XPS.** Escalab 250Xi (ThermoFisher Scientific, United Kingdom) was used to carry out high-resolution photoelectron spectroscopy (XPS) measurements to determine the chemical binding properties of the surface, using a monochromatic Al Kα source and a spot diameter of 650 µm, with charge neutralization by a flood gun. High-resolution spectra were recorded at the energy step size of 0.1 eV at a pass energy of 10 eV. Peak deconvolution and data analysis were performed using Avantage software supplied by the manufacturer. High-resolution C1s, O1s, and N1s spectra for both studied electrodes are presented in Figure S3.

**Theory and DFT modeling protocols.** The atomistic model structures used to validate the electronic and transport properties of the biosensing electrodes were prepared using the molecular builder in Virtual NanoLab, version 2015.2, from QuantumWise A/S. Mulliken charge populations were assigned to each structure and then optimized using Density Functional Theory (DFT) electronic structure calculations with local density approximation (LDA) exchange-correlation and a spin-polarized basis set, along with a conjugate gradient minimization strategy set to converge to a maximum force of 1e-4 eV/Å or an energy difference of 1e-4 eV/Å. Two-probe systems with a scattering region and single-electrode systems with ghost atoms were prepared for IV characterization and electron transport within a fixed chemical potential, respectively. In the former case, the one-electron DFT Hamiltonian and equations were solved self-consistently, followed by setting the non-equilibrium Green’s function (NEGF) formalism, determining the charge density, calculating the effective potential, and calculating the electron current for a bias voltage range using the Landauer–Buttiger approach. The DFT studies reveal high electronic performance of modified BDD electrodes (Supplementary Information S2.1). The electrochemical behavior and low limit of detection may be attributed to the high surface coverage of the viral protein sub-complex adsorbed on the self-assembled monolayer of 4-aminobenzoic acid functionalized BDD surface, as validated from DFTB (density functional tight binding) and MD (molecular dynamics) results included in the Supplementary Information S2.2.

**Production of recombinant M1 in the bacterial expression system.** The M1 gene of Influenza A virus A/England/195/2009 (H1N1) was PCR amplified using plasmid DNA (pPol-M1) as a template and oligonucleotides (For-M1-BamHI 5′ TTGGATCCAGTCTTCTAACCGAGGTCGAA 3′ and Rev-M1-EcoRI 5′ TTTGAATTC CTTGAATCGCTGCATCTGC 3′) as primers. The obtained PCR product of 770 bp was digested with BamHI and EcoRI enzymes and cloned into a commercial vector, pGEX 2TK (GE Healthcare, US). The resulting plasmid, pM1-GST, was verified by restriction analysis and nucleotide sequencing. pM1-GST was used to transform BL21 *E. coli* strain, and the recombinant strain was used to overproduce M1-GST after addition of IPTG (final concentration 1 mM). The 54 kDa protein was visualized on a gel stained with Coomassie Blue and purified by affinity chromatography on glutathione resin (GE Healthcare, US). 0.2 mg of pure M1-GST protein was obtained from 0.25 l of culture.

**S1. Modification of BDD electrodes**

**S1.1 Modification of the BDD surface by the diazonium salt of 4-aminobenzoic acid**

The examples of cyclic voltammograms of reduction and deposition of diazonium salt of 4-aminobenzoic acid in diluted HCl solution at the BDD electrode surface are presented in Figure S1. In the first cycle, an irreversible reduction peak at −.017 V vs. Ag|AgCl was observed. This may correspond to the electroreduction wave of the diazonium salt and the production of an aryl radical which in the following step forms a covalent bond on the BDD electrode surface^2^. In the further scans (from 2 to 10), a decreasing current is observed, and in the last scans, the reduction peaks are not present. This proves the surface saturation and suggests that a thin layer of benzoic acid functional groups was obtained.


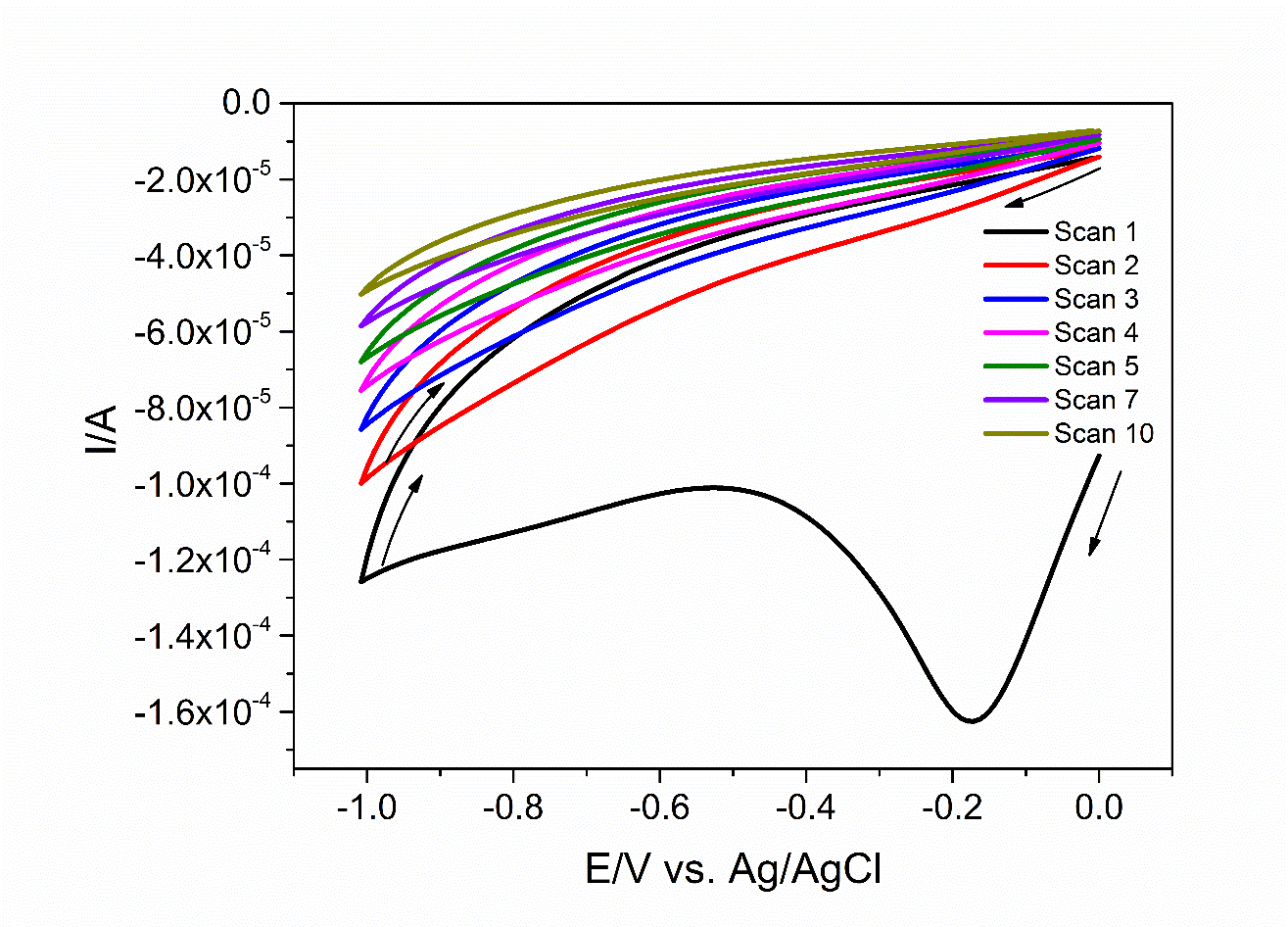


**Figure S1.** Cyclic voltammograms of BDD electrode in contact with diazonium salt of 4-aminobenzoic acid in diluted HCl. Ten cycles performed at a scan rate of 100 mV/s.

**S1.2. Study of composition of modified BDD electrodes**

The X-ray photoemission spectroscopy analysis was carried out to determine the chemical composition of samples functionalized with 4-aminobenzoic acid (BDD-4a) and after subsequent attachment of antibodies to the surface (BDD-aM1) as illustrated in scheme (Figure S2).

**Figure S2.** The electrografting of the BDD surface electrode by electrochemical reduction of the diazonium salt of 4-aminobenzoic acid.

Figure S3a presents a wide energy range spectra for different samples. The main contribution to the signal came from carbon (C1s), oxygen (O1s) and nitrogen (N1s) peaks, as expected. Its total share was over 95%. Silicon, sodium, chlorium and calcium were also present in sample BDD-4a; the 3% share of silicon in the spectra originates from substructure of the electrode while the remaining components were residues from PBS buffer, concentration of neither exceeded 1%. Sample BDD-aM1 also had under 5% contribution from chlorine, sodium and phosphorus. Its origin was similar to that explained above. Wide OA and CA peaks, visible on each spectrum are Auger peaks of oxygen and carbon, respectively.

High-resolution C1s, O1s, and N1s spectra for both studied electrodes are presented in Figure S3b. C1s spectra are composed of four components. Two main components located at approx. 284.3 and 284.9 eV corresponding to C-C bonds on the BDD surface^3,4^. The remaining peaks used for deconvolution of the spectra correspond to C-O or C-N bonds (peak located at 286.0 eV) and C=O bonds (at 288.8 eV). The two oxygen components in the energy range common for organic O-C and O=C bonds were used for deconvolution of O1s spectra. N1s spectra composed of multiple peaks, and it attributed to N-H_3_, N-H_2_, and N=C bonds^5^. A low peak at 407.0 eV was observed from NaNO_2_, and it was used for functionalization of BDD electrode. Binding energies and concentration of all the components used for deconvolution of high-resolution spectra were summarized in Table S1. The presented fitting is in agreement with previous findings^3,5,6^

**Table S1.** Binding energy (BE) and concentration of individual components in high-resolution C1s, N1s and O1s spectra of 4-aminobenzoic acid and M1 antibody protein modified BDD electrodes.

|  | | Sample BDD-4a | | Sample BDD-aM1 | |
| --- | --- | --- | --- | --- | --- |
|  |  | Peak BE [eV] | Atomic % | Peak BE [eV] | Atomic % |
| C1s | C-C_(1)_ | 284.3 | 56.3 | 284.3 | 30.0 |
|  | C-C_(2)_ | 284.9 | 21.8 | 285.0 | 9.1 |
|  | C-O /C-N | 286.0 | 4.0 | 286.2 | 24.0 |
|  | C=N | -- | -- | 287.2 | 0.2 |
|  | C=O | 288.8 | 1.6 | 288.8 | 10.5 |
| O1s | O-C | 532.0 | 11.6 | 531.7 | 3.6 |
|  | O=C /O-N | 533.5 | 2.8 | 532.4 | 9.7 |
| N1s | N-H_2_ | 400.0 | 0.8 | 400.2 | 9.1 |
|  | N-H_3_ | 401.2 | 0.9 | 402.4 | 2.5 |
|  | C=N | -- | -- | 398.1 | 0.1 |
|  | NO_2_ /NO_3_ | 407.0 | 0.2 | -- | -- |


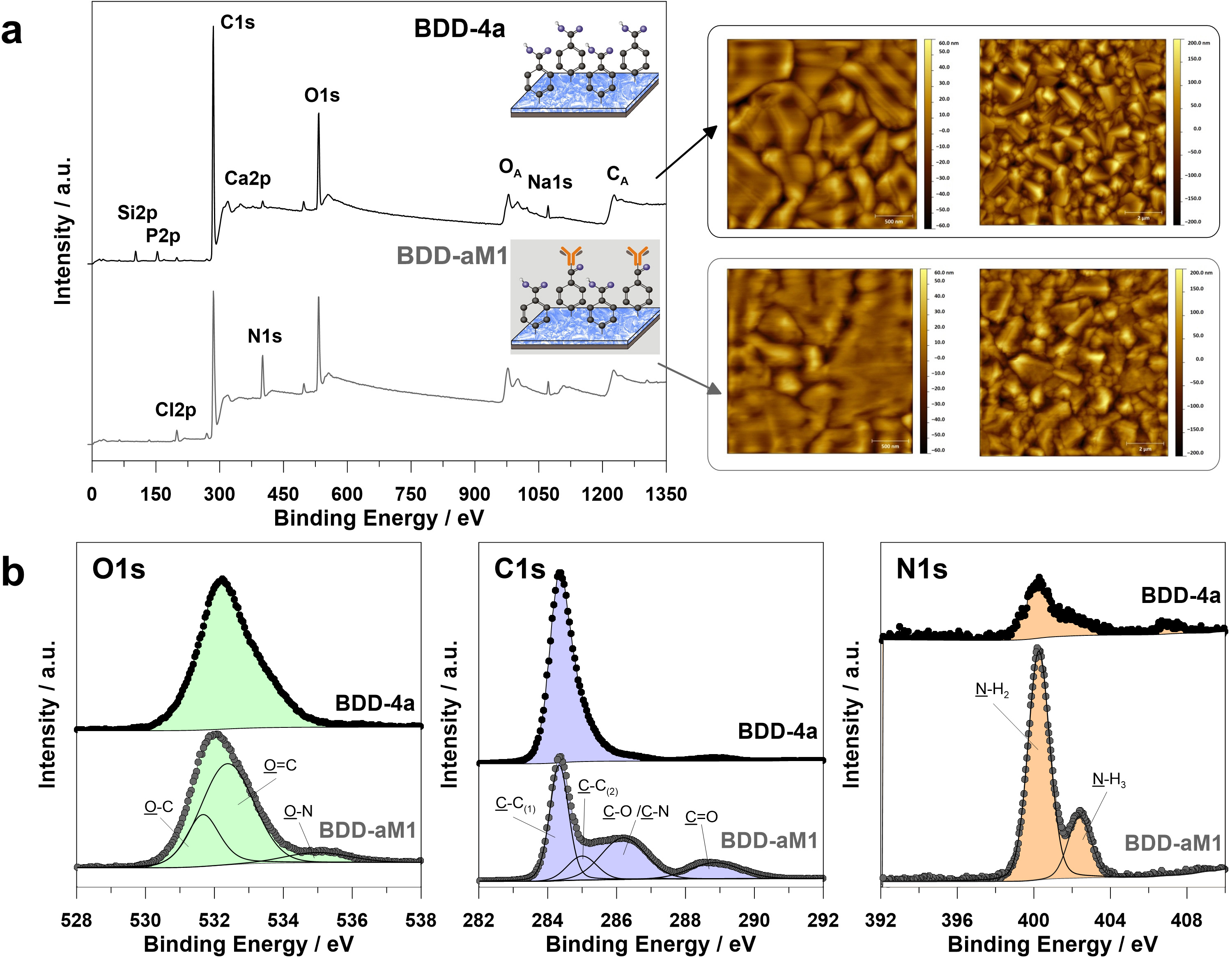


**Figure S3.** AFM morphology along with XPS vs. modification of BDD surface by 4-aminobenzoic acid and M1 antibody protein (a). The high-resolution O1s, C1s and N1s spectra (b).

High-resolution C1s spectra of BDD-aM1 electrode has a much higher share of C-O/C-N component present in the C1s spectra that goes in hand with over a fivefold increase of nitrogen in the registered N1s spectra. Similarly, an increase of oxygen concentration in the energy range of O=C/O-N bonds is observed. This is accompanied by a decreased share of signal originating from the BDD electrode surface, as a result of electrode coverage with a thin film of antibodies.

Next, AFM morphology along with XPS show clear modification of the BDD surface by the antibody protein (Figure S3a). First, the decrease in intensity of C1s spectra was recorded, followed by the expression of additional peaks from C=O or C-N/C-O bonds; the products from the reaction between carboxylated BDD and protein. Second, the N1s peak is much more pronounced, it is conceivable to expect that this increase results from the N-H_3_, N-H_2_, or N=C groups on the antibody surface. The modification is clearly manifested by AFM morphology by the flattening of the valleys and closing of intergrain regions of the BDD surface, whereas the sharp edges of diamond crystallites were smoothed by protein overlayer.

***S1.3 Biosensor for influenza virus detection - stability studies***

In order to verify the electrode stability, BDD-aM1 was kept in PBS solution at 4°C for 24 h. The impedance spectra were registered just after BDD modification by aM1 species and after 24 hours of conditioning.


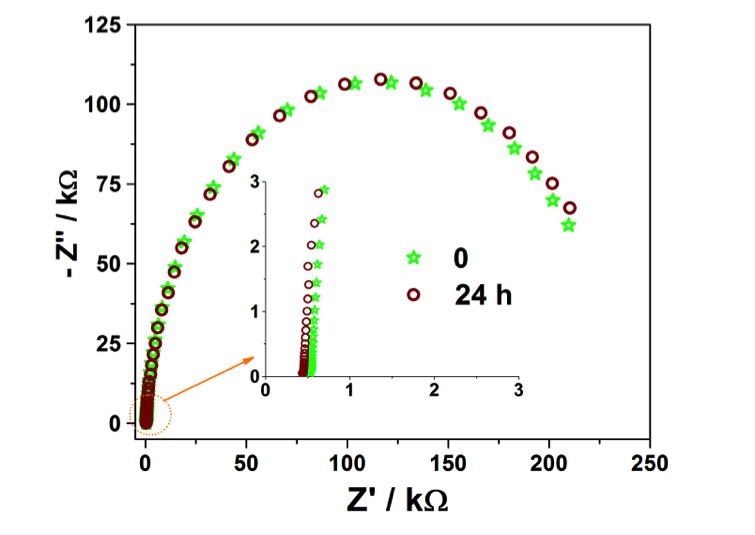


**Figure S4.** The impedance spectra registered for BDD-aM1 electrode at open circuit potential just after BDD modification and after 24h of incubation in PBS buffer.

The spectra were measured in PBS solution without addition of any redox centers so that the electrode does not come in contact with other ion species than buffer. As it could be observed in Fig. S4, the shape of impedance spectra is preserved and proves the electrode durability over 24 hours and is a promise for further commercial application.

**S2. Theory and modeling protocols**

***S2.1. Electronic properties modeling***

First-principles calculations within density functional theory (DFT) method^7^, available within the program Atomistix ToolKit version 2015.2, QuantumWise A/S (www.quantumwise.com)^8,9^ was used for electronic and transport properties of the biosensing electrodes. The local-density approximation (LDA)^10^ was used to describe the exchange–correlation energy functional. For the Brillouin-zone integration, a Monkhorst-Pack scheme sampled with 1×1×50 k-points was utilized^11^, and a single-zeta (SZ) basis set was used with a mesh cutoff at 150 Ry.

Since the polycrystalline BDD electrode fabricated by CVD methods are usually dominated by (111) planes^12^, such a surface has been selected for DFT investigation of electronic properties of biosensing system (see Figure S5ab). Gold (111) electrodes were also studied for reference and comparison.

A 4×4 2×1 reconstructed slab super-cell with six carbon layers was used for all surface models. The reconstructed diamond surface was terminated by hydrogen atoms, and two boron atoms were placed within the second carbon layer from the top surface to approximate the acceptor concentration of 3×10^21^ cm^−3^, as reported in earlier studies^13^. The reference Au cell had the same dimensions. Both surface models were functionalized with specific linker groups, which in turn were bound to selected ligands from the sub-complex peptide of anti-M1 protein.

The 4-aminobenzoic acid and glutaraldehyde were used as a protein linker to BDD and gold electrodes, respectively. The ATCTCCCGCTCTCCGGGTAAA bottom C3H sequence from the heavy chain of the anti-M1 antibody protein was covalently bonded to the –COOH groups in linkers.

In addition, ghost atoms with a vacuum basis set were used in models for description of the charge density extending into the vacuum from all the electrode surfaces^14^. Periodic boundary conditions were used to describe the electronic and transport properties of the biosensing electrodes. A large vacuum layer of 20 Å was added to avoid the interaction between the neighbor slab images. All slabs were relaxed to a local energy minima conformation. The 'two-probe' slab models of the influenza protein sub-complex illustrated in Figure S5 were prepared to determine the I–V characteristics, i.e., electron densities and electron tunneling pathways.


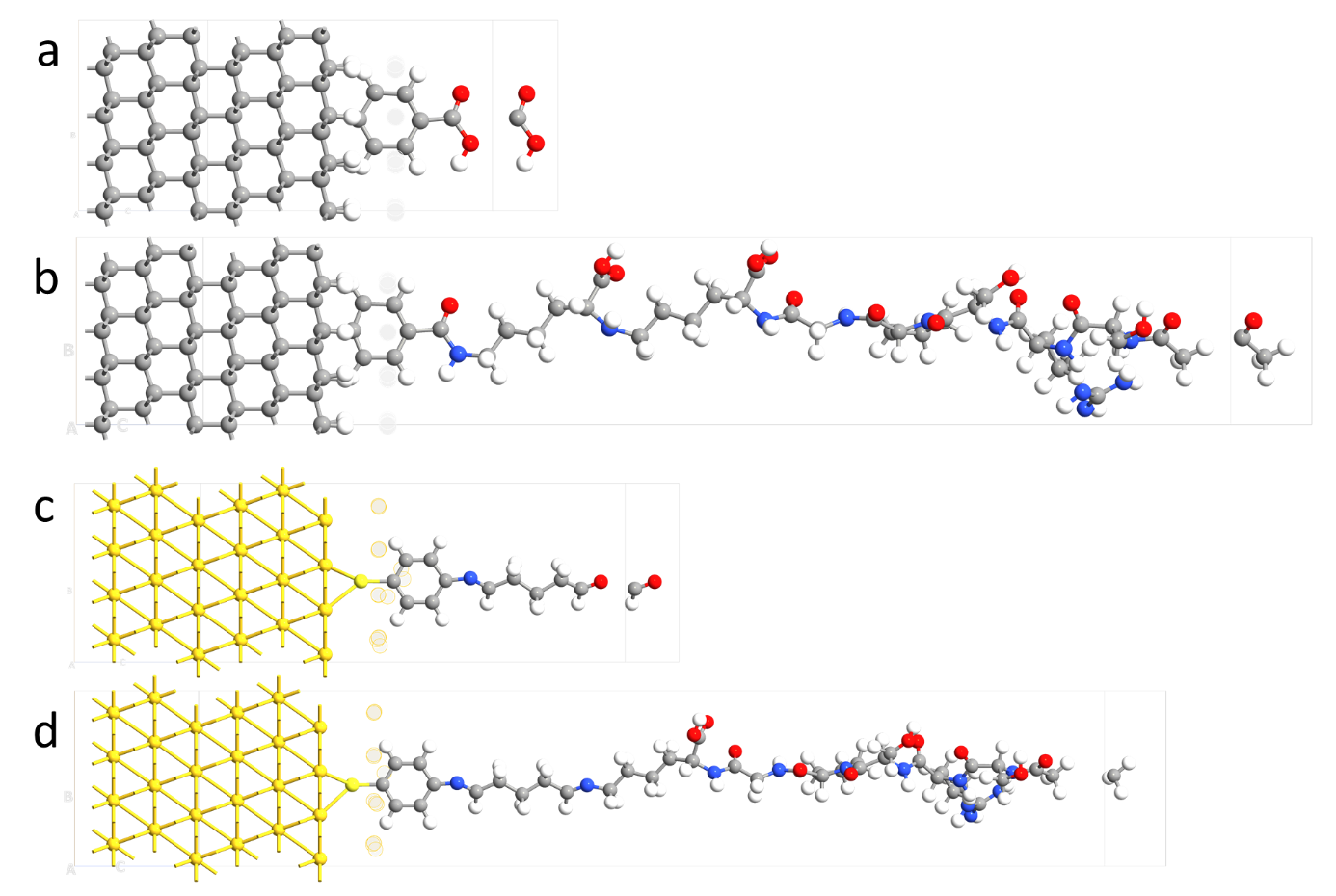


**Figure S5.** Optimized (4×4) model structures of electrodes: H-terminated reconstructed boron-doped diamond (111) and gold (111) surface with adlayer of: (a) 4-aminobenzoic linker, (b) 4-aminobenzoic linker bonded to ligand – part of the anti-M1 antibody protein, (c) glutaraldehyde linker, and (d) glutaraldehyde linker bonded to ligand – part of the anti-M1 antibody protein.

Figure S6 shows the isosurface of the electron-densities difference obtained from a ground state DFT calculation and a constrained DFT calculation. The variation in the electron density in the electrode structures is represented by the yellow clouds, which to a large extent are centered on the carbon or gold top atoms closest to the linkers. The linker-modified electrodes (see Figs. S6a-a1) do not show significant electron density differences, as projected on the linker part when compared to ligand adlayer-modified electrodes (see Figs. S6b-b1).

Ligand functionalized gold electrodes (Figure S6b) show higher electron density differences projected on the plane passing through the top amino acids of ligand complex (right part of slab) than the boron-doped diamond on similar adlayer. The ligand-modified BDD donates electrons to the ligand and to the corresponding protein complexes, resulting in cross-sectional electron tunneling. The corresponding electron pathways reveal relatively high probability of interligand charge transfers^15^. This demonstrates the high probability of electrochemical diffusion to the electrode, thanks to the high in-plane surface charge transfer.


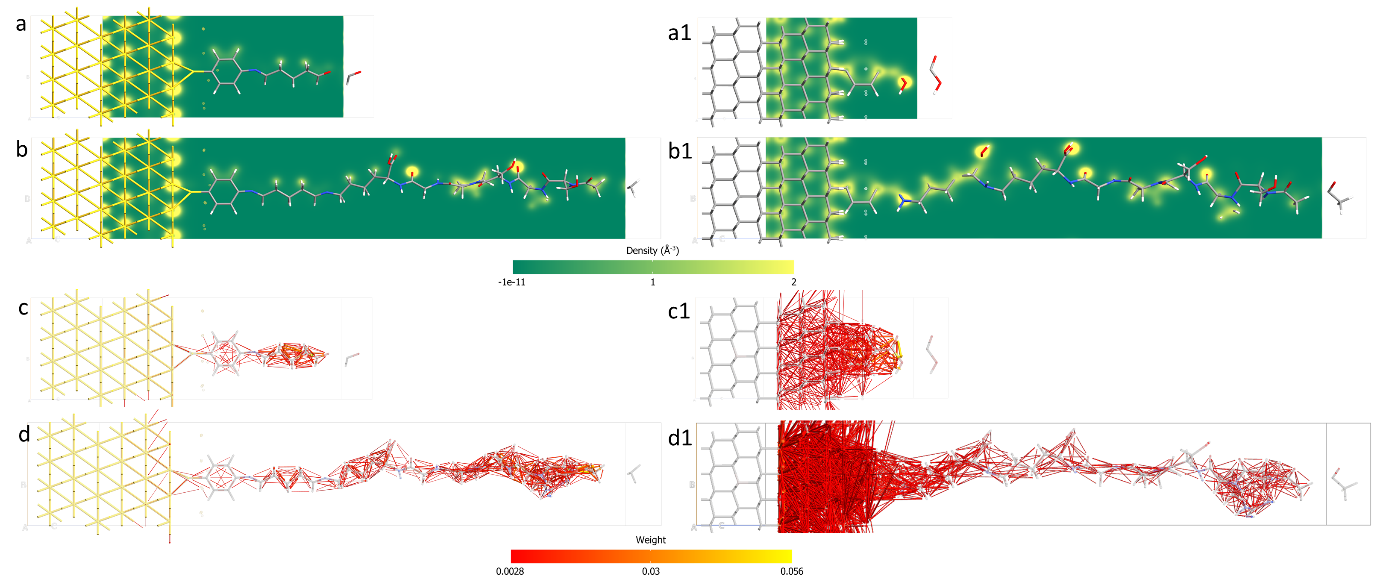


**Figure S6.** Total electron densities distribution maps (ab) and tunneling electron pathways (cd) under a 1V external bias field of Au (left) and boron-doped diamond (right) electrode. The electrodes surfaces with linker and ligand are shown separately.

The I–V curves, shown in Figure S7, were obtained from self-consistent calculations for a bias voltage in the range from 0.0 to 2.0 V. The current in the ohmic region of the transport is higher as the length of the molecule is shorter, as observed in Figure S7. Since the aminobenzoic linker on the BDD electrode is shorter than the glutaraldehyde on the gold surface, the absolute value of the current for the ‘BDD+Linker’ case is almost 5 times higher (see Figure S7a). Unlike the currents through the linkers, the ones including the ligands (see Figure S7b) result in a staircase distribution, which is typical for coherent transport in the strong-bonding regime^16^. Both systems show a minor increase in current up to a certain threshold bias voltage, after which a steep increase is observed due to resonant transmission. The efficient electrode performance may be attributed to the fact that the benzoic group forms a near perfect resonant transmission couple, when compared to DTB molecules^17^, and thus drive significant currents even at low concentrations of the influenza virus.


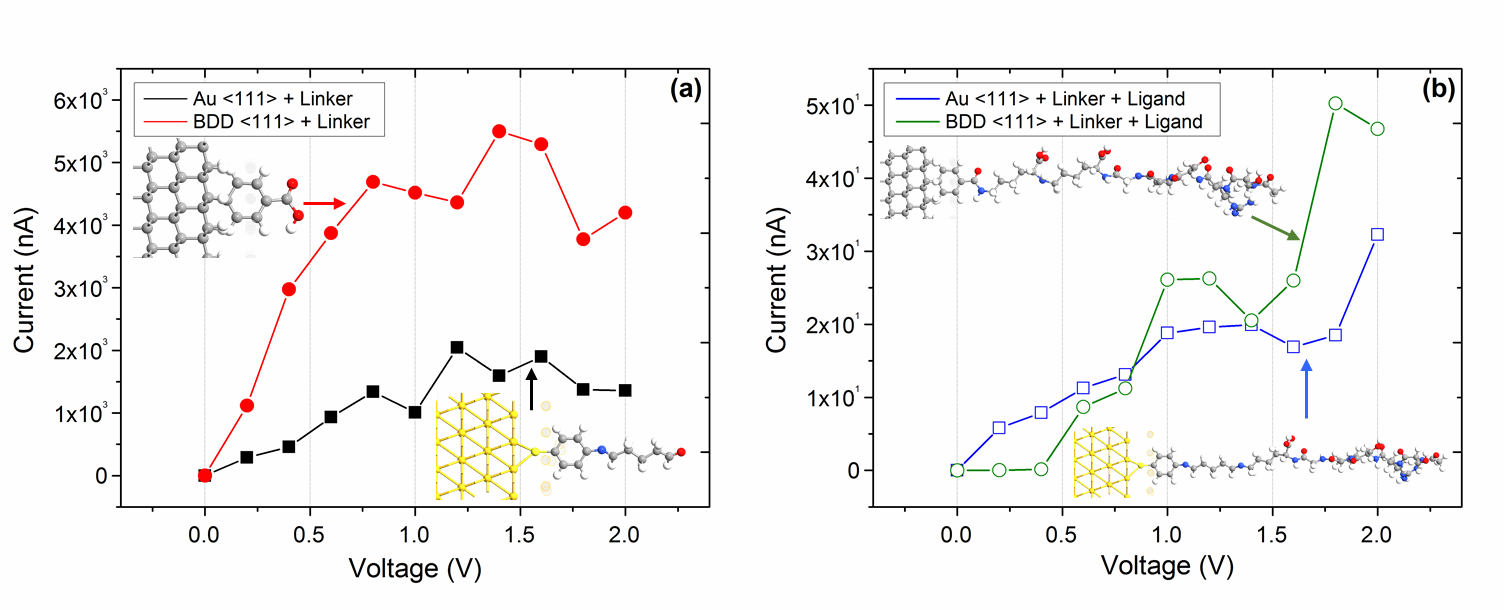


**Figure S7.** Current–voltage (I–V) curves for 'two-probe' systems of the influenza protein sub-complex: (a) BDD and Au electrodes with linker adlayer and (b) BDD and Au electrodes with linker and ligand ad-layer. The inset shows the slab used in the simulations.

On the other hand, since the electrode has a finite (constant) number of electrons, the chemical potential will change during any reactions with the protein sub-complex, because charge transfer from the slab to/from the protein causes the chemical potential to change in the slab. In the real electrochemical experiment, the chemical potential remains constant.  To address this, we used a 'one-probe' approach in which we modeled the initial state of the electrode slab surface with its covalently-bonded linker and the protein molecule in vacuum, and a final state with the absorbed protein molecules on the linker. The electrode side was forced to match bulk-like properties, and the vacuum side to match a gradually decaying boundary condition (0 at vacuum). A Dirichlet boundary condition to normalize the effective potential to zero on the vacuum cell boundary, far from the surface, and the gradient of the potential to vanish on the left of the system (Neumann condition) were used to allow a fixed chemical potential for the electrons and electron transfer from/to the surface region (“true electrochemistry”). The work function for each state was then used to analyze the experimental results. Computed work functions are listed in Table S1.

As can be seen in Table S2, the work function values of electrodes have also become affected by specific modification of surface. There is an observed consequent work function reduction, while the linker and then the ligand adlayer are bonded to the electrode surface. This effect influences charge transfers between the surface bonded to the aM1 antibody and when an influenza protein is bound to it.

**Table S2.** Dependence of the computed work functions (WF) versus specific surface modifications.

| **Modification** | **WF @ Au (111) (eV)** | **WF @ boron-doped diamond (111) (eV)** |
| --- | --- | --- |
| Bare electrode surface | 5.241 | 4.832 |
| Glutaraldehyde/4-aminobenzoic linker | 4.736 | 4.621 |
| Ligand – part of the anti-M1 protein | 3.599 | 4.471 |

***S2.2. Electrode surface coverage model***

To study the most likely concentration of BDD surface coverage by 4-aminobenzoic acid (4-ABA), the surface binding energy as a function of four surface-adsorbed linker molecules was computed for different-sized diamond slab structures (2×2, 4×4, 6×6, 8×8, and 10×10), using Slater–Koster ground-state DFTB calculations with a mio basis set and pair potentials [ATK, www://dftb.org] and room temperature NVT molecular dynamics with a UFF [Rappe and Goddard, J. Ame. Chem. Soc., 114, 10024-10035, 1992] force field to confirm thermodynamic stability of the lowest energy configuration found from the DFTB cases (and closest neighboring configurations). The distance between each of the four adsorbed 4-aminobenzoic acid molecules on the surface varied as a function of the slab size (as shown in Figure S8).

**Figure S8**. Ligand surface area coverage for varying intermolecular distances, as a function of binding energies on a 4×4 surface of 4-aminobenzoic acids.

All slabs were passivated on both sides with H, except on the sites with adsorbed 4-aminobenzoic acid. To compensate for boron doping in the passivated diamond slabs, an external potential with an atomic charge compensation of −0.0171384258539 was used. Energy minimization was performed on all structures, with all but the top layer BDD slab atoms frozen, until an RMS force of 1kcal/Ang, and the single point ground-state binding energies, *E_BIND_*, obtained at each configuration was calculated using *E_BIND_=E_BDD+LINKER_-(E_BDD_+nE_LINKER_)*, where *E_BDD_* corresponds to the BDD slab energy, *E_LINKER_* to the gas phase energy of the linker, and *n* to the number of linker molecules adsorbed (i.e., *n*=4). The optimum 4-ABA coverage on BDD obtained was for the 4×4 structure at an area of 88.14 A^2^ per molecule. The process was repeated for the viral sub-protein complex (referred here simply as ligand) to determine the minimum surface area per ligand of ~793.3 A^2^ on the optimum self-assembled monolayer of 4-ABA.


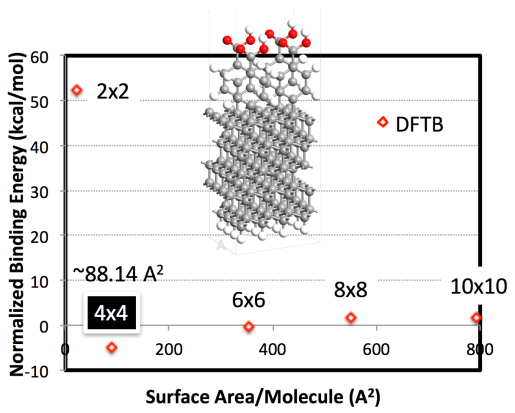


**Figure S9**. (left) Binding energy versus surface coverage for 4-ABA on BDD surface shows minima at 4×4 structure (or ~88.14 A^2^/ligand), and (right) binding energy versus surface coverage for protein ligand on a 4×4 4-ABA surface shows minima at 12×12 (~793.3 A^2^/ligand).

The optimum structures from the DFTB runs (shown at insets in Figure S9) were then used as input for 10ps NVT molecular dynamics ensemble run at a temperature of 298K using LAMMPS^18^ with the Universal Force Field (UFF)^19^. PPrior to the dynamics, converged minimization to 1e-6 energy difference was achieved. Again, the sub-surface slab atoms were frozen to minimize the computational cost. Coverage per molecule was confirmed to be thermally, chemically, and mechanically stable at room temperature. The 12×12 structure containing the four protein ligands on the 4×4 ABA self-assembled BDD layer showed significant conformation changes (ligands coiling on the surface) without explicit solvent, yet the energy minima remained when compared to the 8×8 and 16×16 structures. Explicit solvent interactions are not expected to change the relative binding energies and surface coverage observed in vacuum, yet significantly add to the computational cost.

**REFERENCES**

1. Radhakrishnan, R., Suni, I. I., Bever, C. S. & Hammock, B. D. Impedance Biosensors: Applications to Sustainability and Remaining Technical Challenges. *ACS Sustain. Chem. Eng.* **2,** 1649–1655 (2014).

2. Corgier, B. P., Marquette, C. A. & Blum, L. J. Diazonium−Protein Adducts for Graphite Electrode Microarrays Modification:  Direct and Addressed Electrochemical Immobilization. *J. Am. Chem. Soc.* **127,** 18328–18332 (2005).

3. Ballutaud, D., Simon, N., Girard, H., Rzepka, E. & Bouchet-Fabre, B. Photoelectron spectroscopy of hydrogen at the polycrystalline diamond surface. *Diam. Relat. Mater.* **15,** 716–719 (2006).

4. Ryl, J., Burczyk, L., Bogdanowicz, R., Sobaszek, M. & Darowicki, K. Study on surface termination of boron-doped diamond electrodes under anodic polarization in H2SO4 by means of dynamic impedance technique. *Carbon* **96,** 1093–1105 (2016).

5. Feng, D., Zhou, Z. & Bo, M. An investigation of the thermal degradation of melamine phosphonite by XPS and thermal analysis techniques. *Polym. Degrad. Stab.* **50,** 65–70 (1995).

6. Ryl, J., Bogdanowicz, R., Slepski, P., Sobaszek, M. & Darowicki, K. Dynamic Electrochemical Impedance Spectroscopy (DEIS) as a Tool for Analyzing Surface Oxidation Processes on Boron-Doped Diamond Electrodes. *J. Electrochem. Soc.* **161,** H359–H364 (2014).

7. Sholl, D. & Steckel, J. A. *Density Functional Theory: A Practical Introduction*. (John Wiley & Sons, 2011).

8. Griebel, M. & Hamaekers, J. Molecular dynamics simulations of the elastic moduli of polymer–carbon nanotube composites. *Comput. Methods Appl. Mech. Eng.* **193,** 1773–1788 (2004).

9. Griebel, M., Knapek, S. & Zumbusch, G. *Numerical Simulation in Molecular Dynamics: Numerics, Algorithms, Parallelization, Applications*. (Springer Science & Business Media, 2007).

10. Perdew & Wang, null. Accurate and simple analytic representation of the electron-gas correlation energy. *Phys. Rev. B Condens. Matter* **45,** 13244–13249 (1992).

11. Monkhorst, H. J. & Pack, J. D. Special points for Brillouin-zone integrations. *Phys. Rev. B* **13,** 5188–5192 (1976).

12. Ekimov, E. A. *et al.* Superconductivity in diamond. *Nature* **428,** 542–545 (2004).

13. Zhao, S. & Larsson, K. Theoretical Study of the Energetic Stability and Geometry of Terminated and B-Doped Diamond (111) Surfaces. *J. Phys. Chem. C* **118,** 1944–1957 (2014).

14. Stokbro, K. & Smidstrup, S. Electron transport across a metal-organic interface: Simulations using nonequilibrium Green’s function and density functional theory. *Phys. Rev. B* **88,** 075317 (2013).

15. Strange, M. & Thygesen, K. S. Towards quantitative accuracy in first-principles transport calculations: The GW method applied to alkane/gold junctions. *Beilstein J. Nanotechnol.* **2,** 746–754 (2011).

16. Crljen, Ž., Grigoriev, A., Wendin, G. & Stokbro, K. Nonlinear conductance in molecular devices: Molecular length dependence. *Phys. Rev. B* **71,** 165316 (2005).

17. Stokbro, K., Taylor, J., Brandbyge, M., Mozos, J.-L. & Ordejón, P. Theoretical study of the nonlinear conductance of Di-thiol benzene coupled to Au(1 1 1) surfaces via thiol and thiolate bonds. *Comput. Mater. Sci.* **27,** 151–160 (2003).

18. Plimpton, S. Fast Parallel Algorithms for Short-Range Molecular Dynamics. *J. Comput. Phys.* **117,** 1–19 (1995).

19. Rappe, A. K., Casewit, C. J., Colwell, K. S., Goddard, W. A. & Skiff, W. M. UFF, a full periodic table force field for molecular mechanics and molecular dynamics simulations. *J. Am. Chem. Soc.* **114,** 10024–10035 (1992).
